# Designing and implementing interventions to change clinicians’ practice in the management of uncomplicated malaria: lessons from Cameroon

**DOI:** 10.1186/1475-2875-13-204

**Published:** 2014-05-29

**Authors:** Olivia A Achonduh, Wilfred F Mbacham, Lindsay Mangham-Jefferies, Bonnie Cundill, Clare Chandler, Joelle Pamen-Ngako, Albertine K Lele, Ignatius C Ndong, Sarah N Ndive, Joel N Ambebila, Barnabas B Orang-Ojong, Theresia N Metoh, Mbuh Akindeh-Nji, Virginia Wiseman

**Affiliations:** 1 Laboratory for Public Health Research Biotechnologies, The Biotechnology Centre, University of Yaoundé I, Box 8094, Yaoundé, Cameroon; 2 Department of Global Health and Development, London School of Hygiene and Tropical Medicine, London, UK; 3 Department of Infectious Disease Epidemiology, London School of Hygiene and Tropical Medicine, London, UK; 4 School of Public Health & Community Medicine, University of New South Wales, High St, Kensington, NSW 2052, Australia

**Keywords:** Intervention design, Clinicians, Malaria, Cluster randomized trial, Cameroon

## Abstract

**Background:**

Effective case management of uncomplicated malaria is a fundamental pillar of malaria control. Little is known about the various steps in designing interventions to accompany the roll out of rapid diagnostic tests (RDTs) and artemisinin-based combination therapy (ACT). This study documents the process of designing and implementing interventions to change clinicians’ practice in the management of uncomplicated malaria.

**Methods:**

A literature review combined with formative quantitative and qualitative research were carried out to determine patterns of malaria diagnosis and treatment and to understand how malaria and its treatment are enacted by clinicians. These findings were used, alongside a comprehensive review of previous interventions, to identify possible strategies for changing the behaviour of clinicians when diagnosing and treating uncomplicated malaria. These strategies were discussed with ministry of health representatives and other stakeholders. Two intervention packages - a basic and an enhanced training were outlined, together with logic model to show how each was hypothesized to increase testing for malaria, improve adherence to test results and increase appropriate use of ACT. The basic training targeted clinicians’ knowledge of malaria diagnosis, rapid diagnostic testing and malaria treatment. The enhanced training included additional modules on adapting to change, professionalism and communicating effectively. Modules were delivered using small-group work, card games, drama and role play. Interventions were piloted, adapted and trainers were trained before final implementation.

**Results:**

Ninety-six clinicians from 37 health facilities in Bamenda and Yaounde sites attended either 1-day basic or 3-day enhanced training. The trained clinicians then trained 632 of their peers at their health facilities. Evaluation of the training revealed that 68% of participants receiving the basic and 92% of those receiving the enhanced training strongly agreed that it is not appropriate to prescribe anti-malarials to a patient if they have a negative RDT result.

**Conclusion:**

Formative research was an important first step, and it was valuable to engage stakeholders early in the process. A logic model and literature reviews were useful to identify key elements and mechanisms for behaviour change intervention. An iterative process with feedback loops allowed appropriate development and implementation of the intervention.

**Trial registration:**

ClinicalTrials.gov: NCT01350752.

## Background

Malaria is a major threat to public health and remains the leading cause of death in children under five years in Africa [1]. In Cameroon, malaria accounts for 24% of mortality, 30% of morbidity, with children under five years making up 67% of all malaria-related deaths in the general population [2]. The Cameroon government adopted artemisinin-based combination therapy (ACT) as the first-line treatment for uncomplicated malaria, endorsing artesunate-amodiaquine (ASAQ) in 2004 and adding artemether-lumefantrine (AL) as an alternative ACT in 2006 [3]. The National Malaria Control Programme (NMCP) organized workshops across all regions in Cameroon to inform clinicians at public, mission and private health facilities of the policy change. Effective case management based on parasitological diagnosis and ACT is one of the key strategies for the reduction of malaria burden across the African continent [4].

Malaria rapid diagnostic tests (RDTs) have been presented as a means to realize this key strategy especially in areas where quality microscopy is not available. The Government of Cameroon has adopted this strategy and NMCP is currently developing strategies to scale-up the use of RDTs in health facilities across the country after a pilot study in 2010 [5]. In Cameroon, malaria diagnosis has been reported to be predominantly presumptive leading to over-diagnosis, over-prescription of anti-malarials and under-prescription of ACT [6]. In a study to understand how malaria tests and anti-malarial drugs are used as part of social interactions between clinicians and patients in public and mission health facilities in Cameroon, it was reported that clinicians enacted malaria as a ‘juggling’ exercise, involving attention to pathophysiology of the patient as well as their desires and medical reputations, utilizing tests and medicines for their therapeutic effects as symbols in the process of care [7]. The values and priorities of clinicians in the case management of malaria were in contrast to the evidence-based guidelines recommended by WHO [7]. Hence, the scale-up of RDTs by NMCP could be hindered if appropriate interventions are not designed to address these issues.

Many interventions aimed at addressing the various factors that influence clinicians’ practice in the management of malaria have been developed and implemented. These include interventions aimed at improving the prescription and dispensing practices of clinicians (public and private, formal and informal), and interventions aimed at the purchasing and adherence practices of anti-malarial users and their caretakers [7-16]. However, little is known about the process of designing interventions to accompany the roll-out of malaria RDTs and ACT, including the development of materials and activities for implementation. Widespread deficiencies have been reported in the practices of clinicians in treating febrile patients in health facilities in Cameroon with low levels of adherence to national guidelines, the frequent selection of non-recommended anti-malarials and the use of incorrect dosages. A cluster randomized trial was designed to evaluate the effectiveness and cost-effectiveness of introducing two different clinician training packages, alongside RDTs to equip clinicians with the knowledge and practical skills needed to effectively diagnose and treat febrile patients [17]. The overall aim of the Research on the economics of ACT (REACT) trial was to facilitate optimal use of malaria treatment guidelines through increasing the uptake of RDTs, adherence to both positive and negative RDT results and use of ACT rather than other non-recommended anti-malarial treatments of confirmed malaria cases. This paper documents the processes of design, development and implementation of interventions to change clinicians’ practice in the management of uncomplicated malaria in Cameroon.

## Methods

### Study areas

The study was carried out in Yaoundé and Bamenda in the Centre and Northwest regions of Cameroon, respectively. Yaoundé the capital is predominantly Francophone with an estimated population of 2.5 million, and all seven urban health districts in Yaoundé were included. The Bamenda site encompassed urban Bamenda and five rural health districts within 21-km radius, which serve a predominantly Anglophone population and has an estimated population of 2.0 million. In both sites, facilities included were public district hospitals, primary health centres, mission hospitals, and integrated health centres. Although both study sites lie within the forest ecological zone of Cameroon favourable for the development of the *Plasmodium* parasite and *Anopheles* vector, they have different climatic patterns. The Yaoundé study site has two main seasons: the long wet season that lasts from February to November (with more intense rains between September and November) and a short dry season from December to January and transmission in this site is perennial with an entomological inoculation rate (EIR) of 5.5 infective bites per person per month [18]. The Bamenda study site is characterized by one long rainy season (March to October) and the EIR is similar to that of Yaoundé [18].

### Intervention process

**Figure 1:**
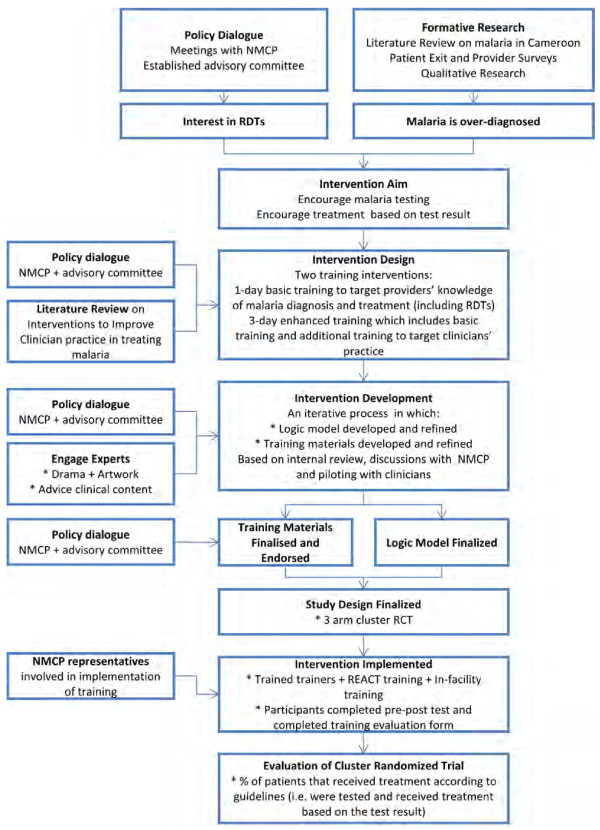
**Intervention design, development and implementation process.** Steps in designing an intervention to improve clinician’s practice in the management of uncomplicated malaria. RCT = Randomized Clinical Trial. NMCP = National Malaria Control Programme. RDT = Rapid diagnostic test. REACT = Research on the economics of artemisinin-based combination therapy.

In the remainder of the paper the process of designing, developing and implementing the intervention and the steps taken at each stage to optimize the process are outlined (Figure 1).

### Literature review on barriers to implementation of malaria case management in Cameroon

In this review, the aim was to outline the factors that compromise the access to and delivery of ACT, and understand clinicians’ practices in the management of uncomplicated malaria in Cameroon. Documents were searched using keywords such as fever, malaria, malaria treatment, malaria case management, anti-malarials and ACT. The review focused on both published and grey literature on Cameroon. The search was carried out in the Ministry of Health, National Malaria Control Programme, University of Yaoundé 1, Catholic University libraries and international organizations including World Health Organization (WHO), United Nations Emergency Fund (UNICEF), German Technical Cooperation (GTZ), Institut de Formation et de Recherche Démographique (IFORD) and the United Nations Development Programme (UNDP). In total, 61 articles were reviewed, consisting of policy documents, research articles from national and international journals, reports, and theses/dissertations written in either French or English.

The review found clinicians’ management of uncomplicated malaria was influenced by: a lack of knowledge of the recommended doses for ACT, pressure to use drugs supplied by the medical boards governing mission facilities, pressure from pharmaceutical marketing agents, community preference of anti-malarial drugs, in-service and pre-service training packages and the availability of drugs in stock. Moreover, the delivery of ACT to health facilities was underlined by a variety of factors including administrative hitches (e g, complex and poorly functioning supply chain), absence of an efficient stock management system, a lack of adequate funding for large scale subsidies and inequity of ACT between public and mission facilities.

### Quantitative formative research

Quantitative formative research was conducted between July and November 2009 to understand malaria case management in Cameroon five years after the adoption of ACT, examine diagnosis and treatment patterns in different types of facility, and investigate factors associated with being prescribed or receiving ACT. A stratified multistage cluster survey was conducted in the two study sites as reported in the study protocol [17]. The quantitative study, carried out through patient exit surveys and clinician/facility surveys, showed that there was over-diagnosis of malaria in health facilities [6]^.^ Although microscopy testing was available in 87% of public and 100% of mission facilities, only 37% of febrile patients were tested for malaria in public facilities and 44% in mission facilities before prescription. Moreover, when patients were tested for malaria 70% of test-negative patients in public and 88% in mission facilities received or were prescribed an anti-malarial. For malaria positive patients, 75.6% in public and 56.7% in mission facilities were prescribed or received an ACT. Eighty-one percent (81%) of public and mission facilities had ACT in stock and 75% of clinicians knew that ACT was the recommended treatment for uncomplicated malaria. Moreover, there were clinicians who knew ACT was recommended but did not consider ACT to be the best anti-malarial for uncomplicated malaria [19]. Clinicians’ preferences over alternative anti-malarials were influenced by their colleagues, their patients and pharmaceutical representatives [19].

### Qualitative formative research

The qualitative component focused on how malaria and its treatment are enacted by clinicians and considered how this relates to emergent evidence-based guidelines. The data were collected between May and June 2010 through focus group discussions (FGDs) with clinicians in the two study sites. Results showed that clinicians enacted malaria as a ‘juggling’ exercise, involving attention to both the pathophysiology and ‘psychology’ of the patient as well as their desires and medical reputations, utilizing tests and medicines for their therapeutic effects as symbols in the process of care. Parasites were rarely mentioned in describing diagnostic decisions [6]. Insight from the qualitative formative research indicated that a supportive intervention should aim to facilitate an adaptation period to encourage troubleshooting with the new tests (RDTs), finding solutions to logistical problems that could be barriers to uptake and a facilitative rather than procedural approach is likely to be most effective. Cost of testing should be either tied into the cost of treatment or removed altogether. A supportive intervention should aim to enable clinicians to confidently explain to patients why testing is important and how the outcome will be different if they are tested compared with not being tested.

### Policy dialogue and establishing aims of the intervention

During the formative phase of this research, discussions with officials from the NMCP revealed that the government was planning to introduce RDTs in 50 pilot health districts in the country. In collaboration with the Ministry of Health (MoH), advisory committees were established in each study site comprising regional delegates for health, malaria officers, policy experts, sociologists, and health economists. These committees were a forum through which the results of the formative research could be fed directly into the implementation strategies of MoH to facilitate a successful uptake of RDTs in health facilities. Findings from the formative research (Table 1) were presented to policy makers in the NMCP in the MoH, and heads of public health and mission facilities for a discussion on the way forward. Through the literature review on barriers to implementation of malaria case management in Cameroon, it was agreed that there is still significant disparity between the existing coverage and effective use of malaria diagnosis and treatment. Based on insight from the formative research, the NMCP in the MoH and the study team agreed that there was need to address the gap between clinicians’ knowledge and their practice for the cost-effective introduction of RDTs. Thus, supporting interventions aiming to change clinician behaviour needed to not only provide skills in testing and knowledge in treatment regimens but also establish habits and confidence in prescribing treatment based on test result as well as strengthen clinician-patient communication to reduce practice based on perceived patient expectations.

**Table 1:** Lessons learnt from literature reviews and formative research

**Literature reviews**		**Formative research**	
**Barriers to the implementation of malaria case management in Cameroon**	**Interventions to improve clinicians’ practice in treating uncomplicated malaria**	**Quantitative**	**Qualitative**
• Lack of knowledge of the recommended doses for ACT	• Positive effect on presumptive treatment of febrile patients, and the accuracy of the doses and advice given	• Malaria prevalence	• Heterogeneous definition of malaria
• Pressure to use supplied drugs from governing medical boards of mission facilities	• Provision of RDTs and training on diagnostic tests led to improvements in the appropriate treatment of malaria	• Over-prescription of anti-malarials	• Heterogeneous treatment for malaria
• Pressure from pharmaceutical marketing agents, providers and community preference of anti-malarial drugs,	• Difficult to draw conclusions from the economic interventions given the limitations of the study design and data available	• Reliance on presumptive diagnosis	• Malaria is an acceptable disease.
• Few in-service and pre-service training packages and the availability of drug in stock	• None of the studies compared the implementation of an intervention across public and private sector providers	• Availability of testing and ACT	• Broad role of anti-malarials
• Delivery of ACT to health facilities governed by a plethora of factors ranging from administrative hitches to the absence of an efficient monitoring and evaluation system			• Discrete roles of malaria tests
• Lack of adequate funding for large scale subsidies and inequity in the subsidization rate of ACT between public and private facilities			

Legend: Lessons learnt from literature reviews and formative research during the process of designing an intervention to improve clinicians’ practice in the management of uncomplicated malaria. ACT = artemisinin based combination therapy.

### Literature review on interventions to improve clinicians’ practice in treating uncomplicated malaria

In a second literature review, the aim was to identify the range of interventions that had been used to improve clinicians’ ability to deliver malaria treatment. A comprehensive search of the published literature was undertaken using Medline, Embase, Global Health, International Bibliography of Social Sciences (IBSS), Centre for Agriculture and Biosciences (CAB) Abstracts and International Network for the Rational Use of Drugs (INRUD) databases. Four concepts (malaria, treatment, intervention, and provider) underpinned the search. Studies were eligible for review if they met all of the following inclusion criteria: i) the study reported on an intervention that was intended to improve the ability of clinicians to diagnose or deliver treatment for uncomplicated malaria; ii) the population exposed to the intervention were clinicians; iii) the study design was a cluster randomized control trial, pre-post design with or without a control group, repeated cross-sectional studies, or a post-only evaluation that had a comparison group; and, iv) the study reported the impact of the intervention on malaria-related outcomes. A total of 1,918 publications were identified once duplicates were removed. Twenty-eight publications met the eligibility criteria and these evaluated 33 different interventions. In the majority of studies the intervention comprised training or an alternative educational process intended to enhance the knowledge and skills of clinicians in treating febrile patients, either specifically in the context of malaria or for a wider range of childhood illnesses. In the context of artemisinin-based medicines, the emphasis shifted to improving malaria diagnosis in the public sector facilities and enhancing the availability and affordability of ACT in the private sector. Most interventions had a positive effect on presumptive treatment of febrile patients, and the accuracy of the doses and advice given. The results also show that the provision of RDTs and training on diagnostic tests led to improvements in the appropriate treatment of malaria, with reductions in the proportions of patients receiving an anti-malarial if they were found to be test negative. Despite the reductions, the proportions of patients that were test negative and received an anti-malarial were still relatively high, suggesting that more would be needed to prevent inappropriate treatment with anti-malarials in patients tested and found not to have malaria parasites.

The review provided valuable background information on what approaches have been tried and tested, as well as the methods used to evaluate their impact. The review also highlighted areas for further work. For instance, while it was shown that provider training and other educational processes can have a significant effect on the knowledge and practices of health workers, the evaluations did not enable conclusions to be drawn about the configuration of a training package. Dimensions that might benefit from further consideration were the length of the programme, learning techniques, importance of supervision and benefits of refresher training. The review also indicated that further work on interventions to improve the appropriate treatment of febrile patients would be valuable. The studies showed that provider training and the provision of RDTs can be beneficial, though suggested that further intervention would be needed to have a substantial effect. Further economic evaluation of interventions was also needed.

### Intervention design

The study team identified a need for the trial to evaluate two scenarios, based on the underlying hypothesis that making RDTs available in public and mission health facilities and providing health workers with some instructions on how to use them (‘Basic Training’) would have a positive impact on the treatment received by patients, but the effect would be greater if the RDTs were accompanied by a clinician intervention package (‘Enhanced Training’) that focused on changing clinician practice. Basic training (BT) was a pragmatic approach, using a standard method to introduce RDTs and improve the knowledge of clinicians on malaria management. In contrast, enhanced training (ET) was designed to go beyond the BT to try and change behaviour as well as improve knowledge. The two interventions were to be compared with routine care in a three-arm cluster randomized trial, which has been described elsewhere [17]. The primary outcome for the trial was the proportion of patients attending facilities that report a fever or suspected malaria and receive treatment according to malaria guidelines.

### Intervention development

#### Logic models

**Figure 2:**
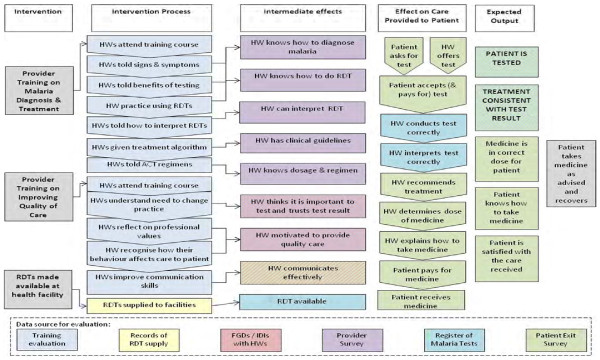
**Logic models.** Logical framework of an intervention to change clinicians’ practice in the management of uncomplicated malaria. HW = Health Worker (Clinician). RDT = Rapid diagnostic test. FGDs = Focus group discussions. IDIs = In-depth interviews.

The underlying research hypothesis for the selection and design of the interventions was summarized in the logic model (Figure 2). This shows the intervention, intervention process, immediate effects, effect on care provided to patients, expected output and expected outcome. It was hypothesized that the basic training will improve clinicians’ knowledge, while the enhanced training targets behaviour as it seeks to reinforce their understanding of the guidelines, give them confidence to use RDTs and motivate them to provide good quality care. The intervention was designed to have intermediate effects that positively affect the process of care, and lead to measurable outputs including whether the patient is tested and whether the treatment is consistent with the test result. Other outputs included whether the treatment is in the correct dose, whether the patient (or their caregiver) knows how the medicine should be taken, and whether the patient is satisfied with the care received. The expected outcome is that the patient recovers having taken treatment as advised.

### Engagement of experts and multiple techniques

With the aims of the intervention defined, malaria intervention experts, policy experts, drama experts, and artists were engaged to assist in developing the various activities of the interventions and methods of delivery. The training modules and activities were designed to address key issues arising from the formative research (Table 2). For the BT arm, a training programme for malaria diagnosis and treatment (with a focus on RDTs) was developed, with training manuals and job aids on how to use an RDT and on the treatment algorithm. Training programmes, manuals and job aids developed in Tanzania [20], Uganda [21] and USAID/WHO [22] were adapted with some modifications to suit the Cameroonian context. Job aids (on how to use an RDT and the malaria treatment algorithm) were provided to facilitate the learning process and to find solutions to logistical problems that could hinder the acceptability of RDTs. Training methods for the BT arm included lectures, case studies/group activities, and a practical session on RDTs where participants had hands-on training in the use of RDTs for the diagnosis of malaria. The BT was designed to last for one day.

**Table 2:** Intervention techniques

**Intervention arms**			
**Basic training**		**Enhanced training**	
**Associated modules and sessions**	**Activities**	**Associated modules and sessions**	**Activities**
**Module 1. Malaria diagnosis**		**Module 1. Malaria diagnosis**	
• Symptomatic diagnosis	✓Pre- and post-quiz on diagnosis and treatment	• Symptomatic diagnosis	✓Pre- and post-quiz on diagnosis and treatment
• Parasitological diagnosis	✓Case studies on diagnosis and treatment	• Parasitological diagnosis	✓Case studies on diagnosis and treatment
**Module 2. Rapid diagnostic testing**		**Module 2. Rapid diagnostic testing**	✓Practical exercise on the use of RDTs
• What is an RDT?	✓Practical exercise on the use of RDTs	• What is an RDT?	✓Case studies on diagnosis and treatment
• Advantages and disadvantages of RDTs compared to microscopy		• Advantages and disadvantages of RDTs compared to microscopy	
• How to use an RDT		• How to use an RDT	✓Case studies on why test for malaria
**Module 3. Malaria Treatment**		**Module 3. Malaria Treatment**	✓Worksheets on RDTs *vs* microscopy
• Treatment algorithm		• Treatment algorithm	✓Testimonial on use of RDTs and discussion
• Treatment when test is negative		• Treatment when test is negative	✓Drama 1- RDT testing before treatment
• Treatment in special cases		• Treatment in special cases	✓Card game - Appropriate Treatment
		**Module 4. Adapting to Change**	
		• Feel comfortable about change in malaria guidelines	✓Drama 2 - Consequences of not prescribing the recommended treatment for uncomplicated malaria
		• Encourage use of diagnostic testing using RDTs	✓Picture scenario/card game- Clinician’s behaviour
		• Encourage the use of ACT for treating uncomplicated malaria	✓Reflecting on quotes- patient perception of malaria and discussion
		• Encourage appropriate treatment based on test results	✓
		**Module 5: Professionalism**	
		• Why is a clinician’s behaviour important?	
		• Encourage clinicians to work effectively with colleagues	
		• Encourage clinicians to provide quality care to patients	
		**Module 6: Communicating effectively**	
		• Understand patients’ perceptions of malaria and treatment in order to communicate more effectively with patients	
		• Develop and improve skills on how to communicate with patients	

Legend: Techniques used to address issues identified during formative research in intervention to improve clinicians’ practice in the management of uncomplicated malaria. ACT = artemisinin-based combination therapy; RDT = rapid diagnostic test.

**Figure 3:**
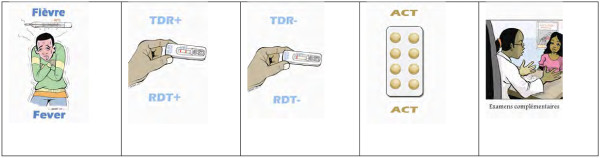
**Appropriate treatment card game.** Rules of the game (3 to 5 players). Step 1: Deal 3 cards per person and place the remaining in a pile face down. Step 2: The first player picks up one card from the pile and then determines if he can give “appropriate treatment” using the correct combination of 3 cards (Fever & RDT positive & ACT OR Fever & RDT negative & Further investigation). If he/she can, the player places 3 cards on the table. If not, his/her turn ends. Step 3: Play passes to the next player and follows from step 2. Step 4: Play continues until someone has given appropriate treatment to five patients. Fever = Patient has a fever. RDT + = Rapid diagnostic test is positive. RDT- = Rapid diagnostic test is negative. ACT = Artemisinin-based combination therapy. Examen Complementaire = Further Investigation.

The enhanced training intervention was designed to change behaviour in the management of uncomplicated malaria thereby acknowledging that knowledge alone does not necessarily change behaviour [7]. The formative research showed that clinicians often know that ACT is the recommended treatment but some prefer to use quinine instead [19]^.^ Similarly, clinicians may know that it is not necessary to give an anti-malarial if the test result is negative, but they do so anyway for other reasons [6]. Therefore the approach was to develop activities that would make the clinicians feel more comfortable about prescribing treatment based on the test result. Multiple techniques (case studies, card game, testimonials, picture scenarios, self-developed participatory drama) were used to form new habits and increase the confidence of clinicians in using RDTs and ACT with respect to each of the issues arising from the formative research (Table 2). A card game on appropriate treatment was developed to improve clinicians’ understanding of the treatment algorithm and encourage them to prescribe in line with test results. The game comprised a pack of five types of card (Figure 3): ‘Patient with fever’, ‘RDT is positive’, ‘RDT is negative’, ‘ACT’, ‘Further investigation’. Players score a point if he/she can provide appropriate treatment for the febrile patient by correctly combining three cards (‘Patient with fever’ card and ‘RDT is positive’ and ‘ACT’ card, or ‘Patient with fever’ and ‘RDT is negative’ and ‘Further investigation’). Play continues until a participant has given appropriate treatment to five patients.

### Ministry of Health approval and piloting

The full intervention package consisting of manuals for facilitators and participants, job aids, card games, drama, case studies, picture scenarios, and table-top flip charts were produced. These intervention materials, together with PowerPoint presentations and standard operating procedures, were presented to NMCP, policy makers of MoH, heads of mission and public facilities for discussion. At the end of the discussion NMCP in the MoH endorsed the training materials and gave permission for the training to go ahead. They also advised on choice of RDT kits, the method of supply of RDTs and the cost per patient (no national policy on RDTs existed at the time of the intervention), number and cadres of clinicians to be trained per facility and the need to produce all the documents in the two official languages (English and French) of Cameroon.

The intervention package was piloted in Buea Health District (not one of the study sites) to validate all study materials and activities. Buea is located in South West Cameroon, a forest ecological zone where malaria transmission is perennial and therefore similar to the areas of the intervention in the trial. Clinicians from public and mission health facilities attended one-day or three-day training on BT and ET, respectively. The intervention package was evaluated by clinicians and their feedback used to refine the package prior to large-scale implementation.

### Intervention implementation

#### Training of trainers

A training of trainers’ workshop was organized to train facilitators on how to use the intervention package to deliver the activities. The ten facilitators made up of trainers nominated by NMCP, theatre experts and study team members also received comprehensive lecture and practical exercises on communication with adults, public speaking, adults and their learning habits, role of an animator, and conflict resolution. At the end of of the training, each facilitator was given a hard copy of the facilitator’s manuals, powerpoint presentations for BT and ET. Faciliators were trained on the standard operating procedures (SOPs) that were developed for each activity, such as how to deliver a workshop, record implementation process, and coordinate a drama and card game. This helped to ensure uniformity in the delivery of the intervention package.

### Workshops

Training workshops were organized simultaneously in Bamenda and Yaoundé sites from 7–11 June, 2011 and each facility was invited to send four clinicians from the following cadre: medical doctor, nurse, pharmacy attendant, and laboratory technician. Ninety-six clinicians from 37 public and mission health facilities attended either a one-day basic training on ‘Ensuring Appropriate Treatment for Uncomplicated Malaria’ or a three-day enhanced training on ‘Ensuring Appropriate Treatment for Uncomplicated Malaria’ plus ‘Improving Quality of Care for Management of Suspected Malaria’. All facilities were represented for the full duration of the training by an average of three clinicians (range of one to four per facility). Of the 111 clinicians invited across the Bamenda and Yaoundé sites, 96 (86.5%) attended the training. Participants were comprised of 60 (62.5%) nurses, 22 (22.9%) medical doctors and 14 (14.6%) laboratory technicians from 37 health facilities. There were on average 24 participants (range of 21–27 participants) in each training workshop.

### Evaluation of training programmes

The basic and enhanced training was evaluated by participants and facilitators. A pre- and post-quiz on malaria diagnosis and treatment was administered to assess participants’ knowledge before and after the training course. Participants were also provided with evaluation forms where they provided their opinions on the general conduct of the training, the activities, and its usefulness in improving their knowledge and feedback on each training module. Training materials are available at [23]. The facilitator also provided feedback on the delivery of each training module and achievement of goals.

Knowledge increase on malaria diagnosis and treatment between pre- and post-training test was 24% in both arms for both sites. A total 68 and 92% of participants in BT and ET, respectively strongly agreed that “it is not appropriate to prescribe anti-malarials to a patient if they have a negative RDT result”. Participants’ feedback showed a strong appreciation for the commitment of the facilitators and well-delivered workshops. Facilitators’ average score for participants’ achievement of learning objectives for each training module was 93.1% for BT and ET in both sites. In both study sites there were questions and concerns throughout the modules on specific aspects of the material but it was felt that almost all of these were resolved by the end of the training.

### In-facility dissemination

At the end of the training course, each facility was provided with training manuals and a CDRom with copies of the presentations, table flip charts, job aids for on how to use RDTs and the treatment algorithm, and a box of RDTs. Participants were also strongly encouraged to train their peers using the training materials within one month after the training course. This was intended to replicate a real life scenario where ‘cascade training’ is the norm. All trained clinicians were issued a certificate of participation. In-facility training was organized with the support of the study team from June-August 2011. Of the 37 facilities included in the study, 34 (91.9%) organized the training with a total attendance of 632 participants ranging from four to seventy-one participants depending on the size of the facility while three (8.1%) facilities did not organize training.

### Supply of RDTs and supervision

SD-Bioline Malaria Antigen P.f/pan-specific kits, chosen in conjunction with NMCP, were supplied by the study team to facilities in BT and ET from June to November 2011. A total of 100 RDTs were supplied monthly and all facilities were routinely supplied with ACT by the government or mission authorities. Facilities were requested not to charge for use of RDTs in children under five years and advised to charge up to 100CFA (0.2USD) for patients above the age of five years. This was in line with government policy for free malaria treatment for children under five years. Monthly supervisory visits by the study team were made to support clinicians in both arms and to provide possible solutions to any challenges faced.

## Discussion

This paper presents the process of designing, developing and implementing an intervention to change clinicians’ practice in the management of uncomplicated malaria to accompany roll-out of RDTs and ACT. Intervention mapping is reported to be a systematic guide to intervention planning, and planning is crucial to effectiveness [24]. Also, it is reported that interventions based on careful identification of relevant change processes as set out in the logic model are more likely to change behaviour successfully [25]. The systematic approach used in this study involving literature reviews, formative research, engagement with national stakeholders, and definition of parameters for the intervention enabled identification and implemention of behaviour change techniques [26] that were more likely to effectively promote awareness, components of motivation and the skills required to change clinicians’ behaviour in the management of uncomplicated malaria. Literature reviews and formative research provided a picture of current barriers to the effective roll-out of RDTs as well as the types of interventions that have previously been tested. This was a critical first step to developing materials and activities capable of promoting behaviour change in the management of uncomplicated malaria in public and mission health facilities.

National organizations have been promoted as the appropriate channels for communicating research findings and for meeting statutory requirements and general expectations for generating and documenting knowledge use [27]. Early engagement of stakeholders, NMCP managers and policy makers at the Ministry of Health was essential to ensure that the interventions were useful and sustainable in the management of uncomplicated malaria in Cameroon. The early engagement also built policy maker-researcher relationships that were critical for active engagements beyond the project. In addition, this engagement facilitated access to key trainers who could effectively deliver the training as these trainers have been involved in other malaria-related interventions at the NMCP.

Consideration of a range of potentially important change processes is necessary when designing behaviour change interventions [26]. From the formative research it was evident that knowledge does not necessarily translate into behaviour change. The multiple techniques (card games, drama, case studies, and testimonials) used in the ET were designed not only to improve skills but also to create awareness, re-inforce spoken instructions and comprehension, build confidence, enhance memory and recall and to motivate clinicians to give appropriate treatment to malaria patients. Moreover, behaviour change techniques were selected and mapped out in the ET arm to address each of the key issues arising from formative research such as the use of a card game on appropriate treatment to address the issue of malaria over-diagnosis and treatment.

Participatory methods are important to define the information to be delivered, the elaboration of that information, the options for its delivery and its implementation [28]. The participatory process followed in this study through piloting of interventions, training of trainers and evaluation of training programmes allowed the clinicians to contribute significantly to the development of the interventions prior to large-scale implementation.

The organization of in-facility training had some challenges. Although all participants were strongly encouraged to train their peers at their health facilities, three facilities randomized to the BT arm in Yaoundé did not conduct in-facility training and this was reported to be due to change of management during the intervention period. These challenges could affect the overall outcome of the evaluation phase of the intervention.

So little is known, or documented properly, at the moment about the detail of interventions to improve clinical practice that at this point there is a need for investment in this field. But, as studies are underway, like this one, then lessons can be learned which mean that future investment should be less. Some of the findings from the REACT trial in improving clinical practice will be generalizable, For example, the findings that interactive learner-oriented training is effective in this setting echoes findings from continuing medical education studies elsewhere as reviewed by [29] which adds to an existing evidence base and supports generalizability. However, researchers, non-governmental organizations (NGOs) and ministries of health should spend more time on intervention design and it is good practice to underpin their work with an understanding of the prevailing context and develop hypotheses for what should work and why. This is particularly important when new approaches are being developed and tested (often on a small scale) to inform policy decisions. It is also important for researchers (or others writing up evaluations) to make sure the interventions are clearly and thoroughly described to ensure that patients and health professionals receive beneficial interventions and avoid unhelpful or harmful interventions [30].

## Conclusion

Formative research was an important first step in determining treatment patterns, factors associated with being prescribed or receiving an ACT and how malaria and its treatment were enacted by clinicians. It was valuable to engage stakeholders early in the process as this helped to know if the interventions were useful and sustainable in the Cameroon context. Literature reviews plus logic models were useful to identify key elements and mechanisms for behaviour change. Designing an iterative process allowed time to develop and improve elements of the intervention. Seeking feedback from participants was valuable prior to large-scale implementation.

## Competing interests

The authors declare that they have no competing interests.

## Authors’ contributions

VW and WFM secured the funding and were responsible for the overall study design and project management. OAA was responsible for coordination and supervision of fieldwork and contributed to literature review. LMJ led the literature review on interventions, logic model, overall study coordination, and supervised the quantitative survey. WFM, OAA, LMJ, and VW designed the interventions and policy engagements. BC led the statistical design. CC supervised the qualitative research, contributed to study design and logic model. PNJ, AKL, ICN, SNN, JNA, BBOO, and TNM assisted in literature review, collected data and contributed in the development of activities for the intervention. ANM coordinated data entry and management. OAA, VW, CC, LMJ, and WFM wrote the manuscript. All authors read and approved the manuscript before submission.
